# Intra-Abdominal Testicular Seminoma in a Woman with Testicular Feminization Syndrome

**DOI:** 10.1155/2011/592124

**Published:** 2011-08-28

**Authors:** Darshana D. Rasalkar, Bhawan K. Paunipagar, Alex Ng, Fernand M. Lai, Shalini Jain Bagaria

**Affiliations:** ^1^Department of Imaging and Interventional Radiology, Prince of Wales Hospital, The Chinese University of Hong Kong, Ngan Shing Street, Shatin, New Territories, Hong Kong; ^2^Department of Anatomical and Cellular Pathology, Prince of Wales Hospital, The Chinese University of Hong Kong, Ngan Shing Street, Shatin, New Territories, Hong Kong; ^3^Department of Obstetrics and Gynecology, UCMS and GTB Hospital, Dilshad Garden, Delhi 110092, India

## Abstract

We report a case of intra-abdominal testicular tumor in a 36-year-old married lady presenting with chief complaints of primary amenorrhea. The patient was later diagnosed with testicular feminization syndrome, a form of male pseudohermaphroditism. This testicular tumor was histologically proven as seminoma. Due to rarity, imaging findings in patients with testicular feminization syndrome and intraabdominal testicular tumor have been poorly documented. So far, only one case report had described the combined role of CT and MR imaging in intraabdominal testicular sex-cord stromal tumor. To our knowledge, this case is first to document USG and MR imaging in addition to MR spectroscopy features in intraabdominal testicular seminoma.

## 1. Introduction

As many as 1 in 3000 babies are born intersexed [1]. Male pseudohermaphroditism is one of the intersexed type, inherited as a sex-linked recessive disorder ranging from the mild cases with hypospadias, cleft scrotum, persistent urogenital sinus, and undescended testes, to the severe alteration in phenotype resulting in testicular agenesis (Turner's syndrome), or dysgenesis (testicular feminization syndrome). Testicular dysgenesis leads to inhibition of spermatogenesis and dominance of sertoli cells, so-called “feminizing” testis also known as an androgen insensitivity syndrome (AIS) [2]. Thus, these patients despite having a male genotype present as asymptomatic, functionally normal but reproductively sterile females. AIS is caused by mutations in the androgen receptor gene and is associated with abnormal testicular development with an increased risk of germ cell malignancy [3]. The risk of neoplasia is also known in a maldescended testis and often increases as the age advances [4].

## 2. Case History

**Figure 1:**
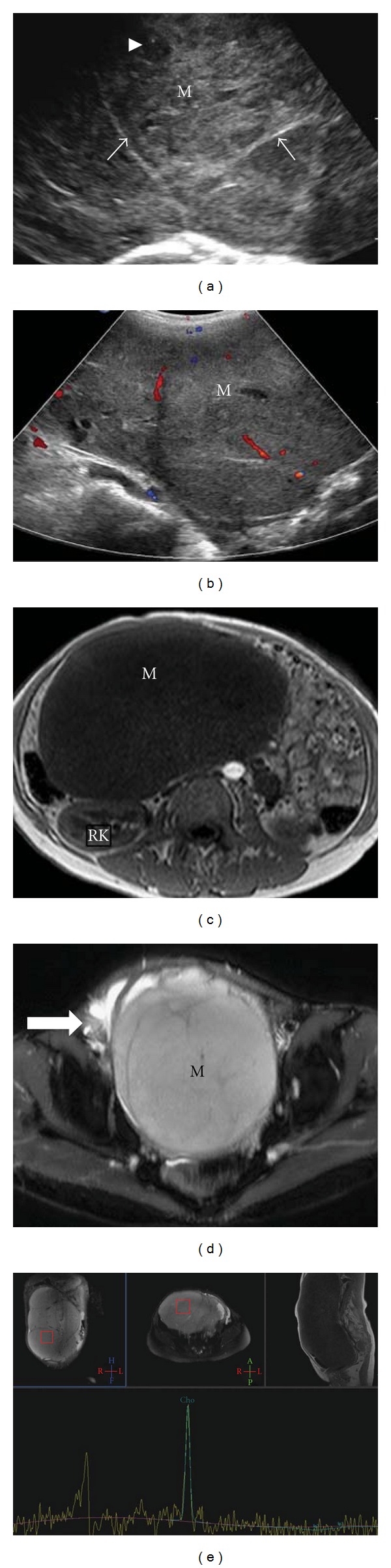
(a) Transabdominal ultrasound showing a heterogeneously hypoechoic mass (M) with internal septations (white arrows) and central necrosis (arrowhead). (b) Color and spectral Doppler images showing few internal vascular channels with arterial flow. (c) T1W axial image of the abdomen. The tumor is hypointense causing mass effect on to the adjacent structures. The right kidney is pushed more posteriorly. (d) T2W axial image showing the tubular gonadal structure in the region of right inguinal canal. (e) MR spectroscopy showing a high choline peak compatible with a tumor spectrum.

**Figure 2:**
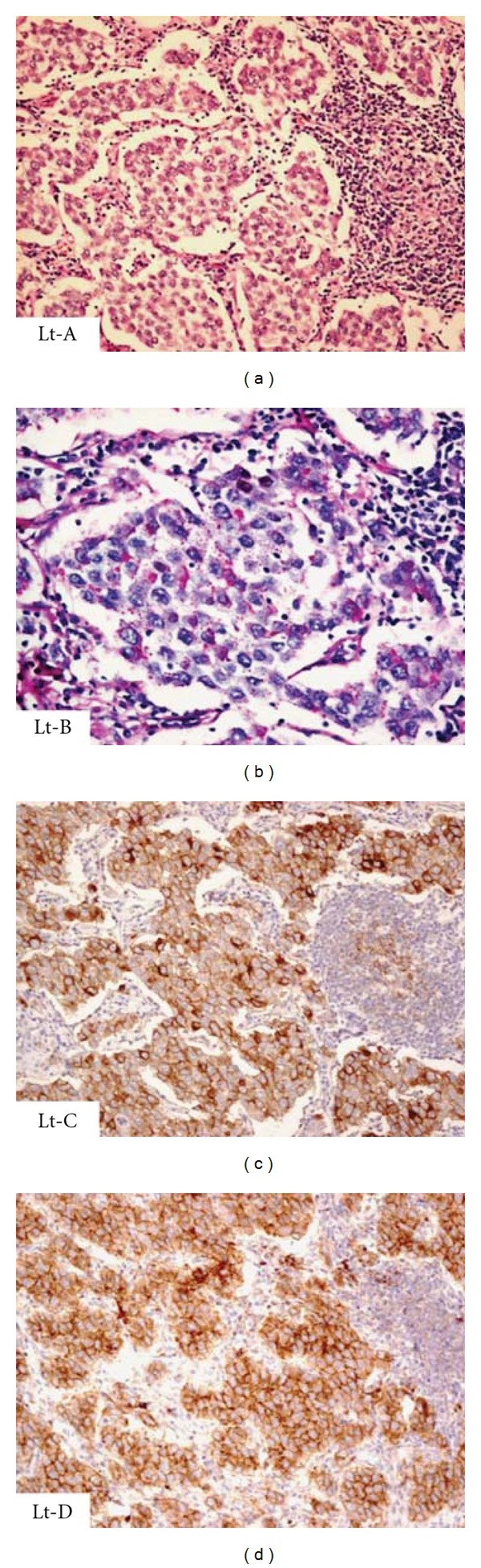
Histology of the left gonad (Lt). (a) classical seminoma, with tumor cells with nests, anastomosing cords, and sheets, ill-formed tubular growth pattern, accompanied by a heavy lymphocytic infiltrate, H&E ×100. (b) Large polygonal tumor cells, with glycogen-rich clear cytoplasm, PAS ×200. (c) Tumor cells are immunoreactive to both placental alkaline phosphatase and to C-Kit (d), both ×100.

**Figure 3:**
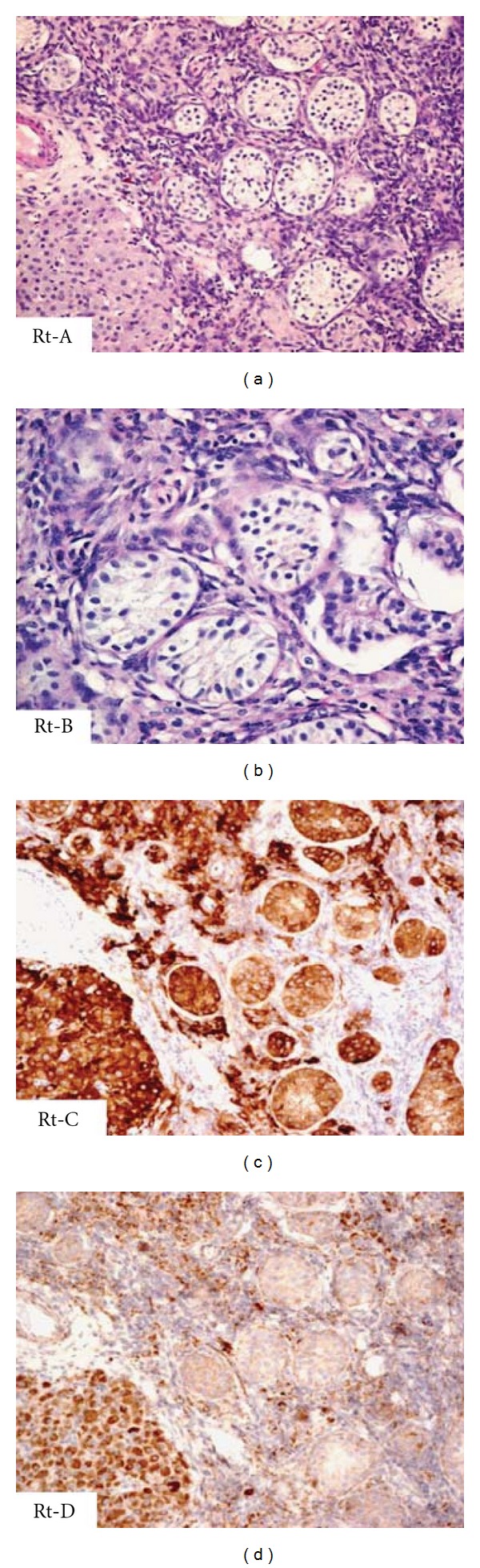
Histology of the right gonad (Rt). (a) Atrophic gonadal composed mainly of tubules of sertoli cells, with absence of germ cells, and the presence of interstitial Leydig cells, H&E ×100. (b) The Sertoli tubules show inactive epithelial cells, absence of germ cells or atypia, H&E ×200. (c) Both Sertoli cells and Leydig cells are immunoreactive to inhibin ×100, while only Leydig cells react to Neuron-Specific Enolase ×100 (d).

A thirty-nine-year old married lady presented with chief complaints of primary amenorrhea. She also had abdominal distension and palpable swelling over the abdominopelvic regions since last two years. There were no constitutional symptoms or weight loss. There was no history of prior biochemical or radiological investigations during her teens. The physical examination revealed normally developed breasts but sparse pubic and axillary hair. On local examination, there was a large firm nontender mass extending over the abdominopelvic regions. Gynecological examinations revealed normal labia; however, vagina was blind (5 cm) with absent cervix. A solid mass was felt at anterior aspect of pelvis. Her hormonal assay revealed raised testosterone levels up to 3.94 (normal levels in women 0.21–2.98 nmol/liter). Tumor markers showed raised beta-HCG levels up to 159 IU/L (normal levels in nonpregnant premenopausal women is less than or equal to 1 IU/L). Carcinoembryonic antigen (CEA) and alpha-fetoprotein (AFP) levels were within normal limits. Blood karyotyping was performed which surprisingly revealed a chromosomal abnormality with 46, XY. Further assessment of mass by an ultrasound showed a large predominantly hypoechoic solid mass measuring 22 cm × 17 cm × 10.56 cm with minimal echogenic internal septations and central necrosis (Figure 1(a)). Few vascular channels were detected on colour Doppler (Figure 1(b)). The uterus was absent and no distinct ovarian tissue identified. On MRI, the mass was homogenously T1 hypointense and T2 hyperintense (Figures 1(c) and 1(d)). Moderate contrast enhancement with central necrosis was identified on postcontrast administration. The right kidney was mildly compressed posteriorly and the aorta, IVC were slightly displaced towards left. A 17 mm long tubular structure with a 14 mm higher signal intensity nodule were detected extending from the right groin to the right inguinal region which were thought to represent a rudimentary gubernaculum of testis +/− undescended testis (Figure 1(d)). The uterus and both ovaries were absent. Overall imaging features were compatible with testicular feminization with possibility of neoplasm arising from cryptorchid intraabdominal type testis. Further MR spectroscopy showed markedly raised choline peak suggestive of tumor spectrum (Figure 1(e)). The other visceral organs were unremarkable. The patient underwent laparotomy removal of pelvic mass and bilateral gonadectomies were performed. Left gonadal tumor was adhered to sigmoid colon and omentum. Right gonad was ectopically situated inside the right inguinal canal. Histopathology of the resected specimen revealed classical seminoma from the left gonad (Figure 2) while right gonad showed presence of inactive sertoli cell tubules with no evidence of malignancy (Figure 3). Omental biopsy and peritoneal fluid were negative for malignant cells.

## 3. Discussion

Intersex disorders result from a genetic defect in chromosomal presentation. They often present with ambiguous external genitalia and on the basis of their gonadal presentation, are categorized into true hermaphrodites and mixed gonadal dysgenesis (pseudohermaphrodites) [5]. Remarkable propensity of the gonads to develop malignant tumors has been documented in 46XY with mixed gonadal dysgenesis and male pseudohermaphroditism [2].

“Feminizing” testis (testicular feminization syndrome), is an inherited sex-linked recessive disorder, a rare form of a male pseudohermaphroditism, characterized by androgen insensitivity resulting from an absence or abnormal cytosol receptor for androgens [6]. Thus, despite possessing a male karyotype (46, XY); phenotypically patient presents as an asymptomatic female usually discovered during perimenarchal stage when the individual fails to menstruate, often presenting in their early 2nd to 3rd decades. Testicular feminization syndrome is rarely diagnosed in the 4th or 5th decades [7]. The testis is often undescended and the ovaries, uterus, fallopian tubes, and upper third of the vagina are typically absent [2, 6]. Externally, the labia majora and minora are usually well formed.

The estimated risk of malignancy in testicular feminization is 5% [8]. In comparison to other intersex disorders, the premalignant risk is relatively low before puberty [2, 7]. However, the overall risk increases in patients older than 30 years and reaches up to 33% in patients above 50 years of age [9]. Gonadectomy/orchidectomy is therefore can be delayed to allow for a natural puberty [3].

Gonadoblastoma is the most commonly seen tumor in dysgenetic gonad [9]. The seminoma-dysgerminoma can also occur and is most commonly seen in streak gonad in mixed gonadal dysgenesis [8]. Rutgers et al. [2] had reported malignant change in their series of 43 patients with testicular feminization in which four (9%) malignant tumors (two seminomas, a germ cell neoplasm, and a malignant sex-cord stromal tumor). Germ cell tumor remains the most common tumor in cryptorchidism as well as in testicular feminization [2]. Ramaswamy et al. [10] reported a large multicystic sex-cord tumor in a testicular feminization syndrome. Imaging features of testicular feminization syndrome are rarely described in the literature. Single imaging report by Karabulut et al. [4] has described CT and MRI imaging features of stromal tumor of the sex cord in a woman with testicular feminization syndrome which showed internal necrotic and haemorrhagic components. We observed mild necrotic components whereas haemorrhages were not seen. Seminomas complicating undescended intraabdominal testes in a man with cryptorchidism are usually well delineated without evidence of hemorrhage or necrosis [11] and sex-cord stromal tumor in intraabdominal testis with testicular feminization syndrome exhibited heterogeneity in both texture and contrast enhancement [4]. Imaging feature in our case were in between (intraabdominal testicular seminomas in cryptorchidism and intraabdominal testicular sex-cord stromal tumor in testicular feminization syndrome) with no and mild heterogeneity in texture and contrast enhancement, respectively.

In conclusion, though definitive diagnosis of testicular feminization syndrome relies on biochemical tests, imaging findings are useful in documenting the absence of mullerian duct derivatives, identifying the location of the undescended testes, and evaluating the accompanying intraabdominal masses. Imaging may also be helpful in providing tissue characteristics of a potential neoplasm arising from the gonads, their accurate extent and staging which are essential for further management.
